# Progress and challenges in potential access to oral health primary care services in Brazil: A population-based panel study with latent transition analysis

**DOI:** 10.1371/journal.pone.0247101

**Published:** 2021-03-16

**Authors:** Ana Graziela Araujo Ribeiro, Rafiza Félix Marão Martins, João Ricardo Nickenig Vissoci, Núbia Cristina da Silva, Thiago Augusto Hernandes Rocha, Rejane Christine de Sousa Queiroz, Aline Sampieri Tonello, Catherine A. Staton, Luiz Augusto Facchini, Erika Bárbara Abreu Fonseca Thomaz

**Affiliations:** 1 Dentistry Department, Graduate Program in Dentistry, Federal University of Maranhão, São Luís, Maranhão, Brasil; 2 Dentistry Department, CEUMA University, São Luís, Maranhão, Brasil; 3 Global Emergency Medicine Innovation and Implementation (GEMINI), Division of Emergency Medicine, Department of Surgery, Duke University School of Medicine, Durham, North Carolina, United States of America; 4 Research Design and Analysis Core (RDAC), Duke Global Health Institute, Durham, North Carolina, United States of America; 5 MAPH Consortium, Belo Horizonte, Minas Gerais, Brasil; 6 MATH Consortium, Belo Horizonte, Minas Gerais, Brasil; 7 Department of Public Health, Federal University of Maranhão, São Luís, Maranhão, Brasil; 8 Department of Public Health, Center of Biological Sciences of Health, Federal University of Maranhão, São Luís, Maranhão, Brasil; 9 Duke Global Health Institute, Durham, North Carolina, United States of America; 10 Department of Social Medicine, Postgraduate Programs in Epidemiology, Nursing and Family Health, Federal University of Pelotas, Pelotas, Rio Grande do Sul, Brasil; 11 Department of Public Health, Graduate Program in Public Health and Dentistry, Federal University of Maranhão, São Luís, Maranhão, Brasil; UFRGS, BRAZIL

## Abstract

**Objective:**

Compared indicators of potential access to oral health services sought in two cycles of the Program for Improvement of Access and Quality of Primary Care (PMAQ-AB), verifying whether the program generated changes in access to oral health services.

**Methods:**

Transitional analysis of latent classes was used to analyze two cross-sections of the external evaluation of the PMAQ-AB (Cycle I: 2011–2012 and Cycle II: 2013–2014), identifying completeness classes for a structure and work process related to oral health. Consider three indicators of structure (presence of a dental surgeon, existence of a dental office and operating at minimum hours) and five of the work process (scheduling every day of the week, home visits, basic dental procedures, scheduling for spontaneous demand and continuation of treatment). Choropleth maps and hotspots were made.

**Results:**

The proportion of elements that had one or more dentist (CD), dental office and operated at minimum hours varied from 65.56% to 67.13 between the two cycles of the PMAQ-AB. The number of teams that made appointments every day of the week increased 8.7% and those that made home visits varied from 44.51% to 52.88%. The reduction in the number of teams that reported guaranteeing the agenda for accommodating spontaneous demand, varying from 62.41% to 60.11% and in the continuity of treatment, varying from 63.41% to 61.11%. For the structure of health requirements, the predominant completeness profile was "Best completeness" in both cycles, comprising 71.0% of the sets at time 1 and 67.0% at time 2. The proportion of teams with "Best completeness" increased by 89.1%, the one with "Worst completeness" increased by 20%, while those with "Average completeness" decreased by 66.3%.

**Conclusion:**

We identified positive changes in the indicators of potential access to oral health services, expanding the users’ ability to use them. However, some access attributes remain unsatisfactory, with organizational barriers persisting.

## Introduction

Since the creation of the Unified Health System ((*SUS*, in Portuguese), Primary Health Care (PHC) has been strengthening as a priority strategy to guarantee population access to health services [1], being the Strategy Family Health (FHS) the main PHC organization model [2].

In the scope of oral health, the Ministry of Health (MS) launched, in 2004, the National Oral Health Policy. This policy advocates the reorganization of oral health care at all levels, with PHC as a priority, through the implementation of oral health teams (OHT) linked to the FHS [3]

Although oral health policies are fundamental to the guarantee of health rights [4], difficulties in accessing these services are a major challenge in Brazil [5–7].

There is evidence that accessibility problems can impact oral health indicators [5, 8], so universal accessibility to PHC has been advocated as a strategy to reduce health inequities [9, 10]. The concept of access, however, is complex and multidimensional [11]; it can be defined as the relationship between the user, their health requirements, and the available health services [12]; it does not equate to the basic use of health services [13].

In this study, we analyze the concept of potential access, characterized by the availability and organization of services and technological resources [14–16] and determined by the presence of factors that enable the use of services, incorporating to this concept the factors that can limit or expand the capacity of use by the individual [15], such as the availability of an adequate structure and organizational aspects of the work process [17].

After almost a decade of implementation of the National Oral Health Policy, Ordinance No. 1,654 of the Ministry of Health created the National Program for Improvement of Access and Quality of Primary Care (*PMAQ-AB*, in Portuguese), with the objective of fostering the increase in access and improving the quality of PHC. One of the stages of the *PMAQ-AB* was the evaluation of the structure of health facilities and the work processes related of PHC teams [18–20]. However, we have not identified any studies that analyze potential access to oral health services, comparing the two evaluations.

It is considered relevant to analyze whether the *PMAQ-AB*, a one of the world’s largest initiatives to improve primary care performance, which collected data to a Brazilian nationwide evaluation program, induced the improvement of potential access to oral health services in Brazil. Our hypothesis is that there was an improvement in access to these services.

The transition analysis of latent classes, a robust statistical method used in this study, allows the evaluation of the behavior of access indicators over time, identifying possible changes in the behavior of these indicators, and thus, subsidizing health policies, guiding the implementation of measures that expand the capacity of use and promote the improvement of the quality of the offered oral health services.

Therefore, the objective of this work is to compare potential access indicators that assess the structure of health services and the work process of PHC teams obtained in the first two assessments of PMAQ-AB, carried out in 2011–2012 (cycle I) and 2012–2014 (cycle II), in Brazil, and, thus, to identify possible changes in the behavior of these indicators.

## Methods

### Design of the study

This is an ecological panel study [21, 22], with data from two national surveys, referring to the Cycles I and II of the external evaluation of the *PMAQ-AB* [23, 24]. For this study, we considered units of analysis from two levels of aggregation, namely, variables of the PHC facilities (structure) and variables of the oral health in PHC teams (work processes)

### Context and period of the study

The *PMAQ-AB* is a program of the Ministry of Health, with voluntary participation by managers and professionals, which promotes a culture of evaluation of primary care public services, encouraging payment for performance. We proposed to implement strategies that qualify these services on the basis of the performance evaluation for participating municipalities that achieve improvement in the quality of services offered to users [18, 25].

The program consists of successive cycles. Each cycle consists of four phases: accession/contractualization, development of actions (cross-sectional phase), external evaluation, and certification/re-contractualization. Cycle I began in 2011, by Ordinance 1,654, which instituted the program within the scope of the *SUS*. External evaluation (made by educational and research institutions in Brazil) occurred in 2012. Cycle II, in turn, began at the end of 2012 and was completed in the first quarter of 2014, and external evaluation was conducted in 2013/2014 [23, 24, 26].

### Participants

When the first cycle of the PMAQ-AB was carried out, there were 43,062 PHC facilities registered in the National Register of Health Care Facilities (*CNES*, in Portuguese) [27]. In this cycle, the *Brazilian Infrastructure Census of the Basic Health Units* was carried out. Thus, all family health units, health posts, health centers, basic health units and outposts registered with CNES in 2012, regardless of adherence to the program, had their structure evaluated [17]. However, only the teams that joined had the work process evaluated.

After excluding the closed and deactivated facilities, as well as those the evaluators were unable to access, a total of 38,812 facilities were evaluated, irrespective to their adherence to the *PMAQ-AB*. In relation to PHC teams, there were 32,337 units at that time. Of these, 17,482 (54.1%) adhered to Cycle I of the program, but 280 (1.6%) were not evaluated due to refusals, absence of the professional at the unit even after several attempts, or the shortcomings of the evaluators to travel to the PHC facilities. These teams were distributed in all 27 Federative Units (FU) and 3,965 (71.2%) Brazilian municipalities.

In Cycle II, there were 45,429 registered facilities [31]. In this cycle, however, only PHC facilities in which at least one team joined the *PMAQ-AB* (n = 24,055) were evaluated. In relation to PHC teams, of the 33,998 teams, 30,541 (89.8%) joined the *PMAQ-AB*, located in 5,007 (89.9%) Brazilian municipalities and in all 27 Federative Units [28, 29].

For our study, all PHC facilities (n = 23,022) and PHC teams (n = 15,699) that adhered to the Cycles I and II of the *PMAQ-AB* were included.

### Location of study

Brazil is the largest country in South America and Latin America, being the fifth largest in the world in a territorial area. It has a population of 190,732,694 inhabitants, distributed in 26 states, a Federal District, and 5,570 municipalities [30]. The country is divided into five regions (north, northeast, midwest, south, and southeast), with the north being the largest in territorial area, however, with the lowest population density (3.77 inhabitants/km^2^) and also with the most inferior gross domestic product (GDP). In contrast, the southeast region, the second smallest in Brazil, is the most populous, most developed area, with a population density of 92.05 inhabitants/km^2^ [30] and with the highest GDP in the country [30].

### Data collection process, study variables, and instruments

To conduct the external evaluation, the Ministry of Health was supported by teaching and research institutions in the organization and development of fieldwork, including selection and training of evaluators who applied the data collection instrument. The evaluators conducted visits to health facilities, performing on-the-spot investigations and interviews with professionals from the teams that joined the PMAQ-AB, including the verification of supporting documents about the actions carried out by the PHC teams. Data collection was performed using tablets, containing a specific application with a standardized instrument, produced through a consensus between peers and previously tested [24, 31].

In cycle I, the external evaluation instrument was organized in three modules and in cycle II, in six [24, 32]. For Cycle I, we used data from Modules I (Observation of the structure of the PHC facilities—general and the dental offices) and II (Interviews with professionals on the work processes related to oral health in PHC teams and examination of documents in the PHC facilities). In Cycle II, we used data related to the structure of health facilities (Module I) and the work process of the oral health team (Modules VI).

Initially, variables related to the structure of the health units and the work process of the PHC teams for oral health were selected, available in the questionnaires of the external evaluation of the PMAQ-AB. The choice of variables to be included in the analysis was based on theoretical criteria, among attributes related to potential access.

For the structure of the health units, variables were selected that reflected elements of human resources, physical structure and organizational structure [17, 33, 34]. For the work process, variables related to the organization of the agenda were selected in order to allow access to the first consultation and the continuity of treatment [35–37], as well as the provision of various health services in the dental office and at home [38, 39].

The selection of variables that would remain in the final model was based on the data, through factor analysis. Variables with small and non-significant factor loads were excluded from the model, selecting the set of variables and latent classes with the best fit.

Thus, the structure variables used were: presence of the dentist in the PHC facilities (Yes or No), presence of the dental office in the PHC facilities (Yes or No), and operation of the PHC facilities, for at least five days a week in the morning and afternoon shifts (Yes or No). The variables of the work processes were schedule appointments every day of the week (Yes or No); perform basic procedures, including topical application of fluoride, amalgam restoration, composite resin restoration, extraction, delay curative/access to the pulp or pulpotomy, tooth alveolar abscess drainage, scaling, smoothing, and supragingival polishing (Yes or No); performs home visits (Yes or No); organize the agenda to ensure attention to spontaneous demand (Yes or No); and organize the agenda to ensure continuity of treatment (Yes or No).

### Statistical analysis

We estimate the absolute frequencies and percentages of each variable in the study using the STATA® statistical package, version 14.0. The Chi-square test was used to identify differences in prevalence between the two periods (Cycles I and II).

Latent transition analysis (LTA) was used to identify classes of completeness of the structure of health facilities and adequacy of the work processes of the PHC teams and to model the transition between these classes over time. LTA is a longitudinal version of Latent Class Analysis (LCA) [40, 41]. LTA is a robust statistical approach used to identify underlying subgroups in a population, which share similar characteristics, based on a number of observed categorical variables [41, 42]. The LTA considers that the units of analysis can change latent class over time; therefore, in LTA, the terminology “latent status” is adopted instead of “latent class.” For this analysis, the statistical modeling program MPlus®, version 8.0, was used.

The first step of LTA was the selection of the number of latent classes/statuses. For this purpose, models were prepared with different numbers of latent classes/statuses and the ones with the best fit quality were selected according to the statistical test of likelihood ratio, p-values, degrees of freedom (df), Akaike information criterion (AIC), bank identifier code (BIC), and entropy for models with two to four latent statuses. Although p-values were reported for all models, they were not considered for those with high df (df ≥ 99) [41, 43, 44]. Low AIC and BIC values reflect a better balance between model fit and parsimony. On the basis of these criteria, we confined the model selection to models of three statuses. Entropy with values close to 1 indicates a clear design of classes [45]. Two models were created: one for the structure of health facilities and one for the work process of PHC teams, independently.

In all, three groups of parameters are estimated in LTA, namely, estimation of the probabilities of the characteristics of units belonging to a latent class/status, for example, when modeling the characteristics of the structure of the PHC facilities over time, these probabilities reflect the proportion of facilities with certain characteristics that belong to each latent class/status; prevalence of observations in each latent class/status at each time; and estimation of transition probabilities between latent classes/statuses over time [41, 46].

In the LTA, multiple aspects of a phenomenon can be accessed over time to jointly indicate the latent class/status. Therefore, within each period, the outcome studied can be modeled as a multivariate phenomenon [47].

Choropleth maps describing the distribution of latent transition patterns to Brazilian FU were prepared through the ArcGIS® program, version 14.0 (California, United States). In all, five Choropleth maps were prepared for the structure, and another five were prepared for the work processes.

**Fig 1:**
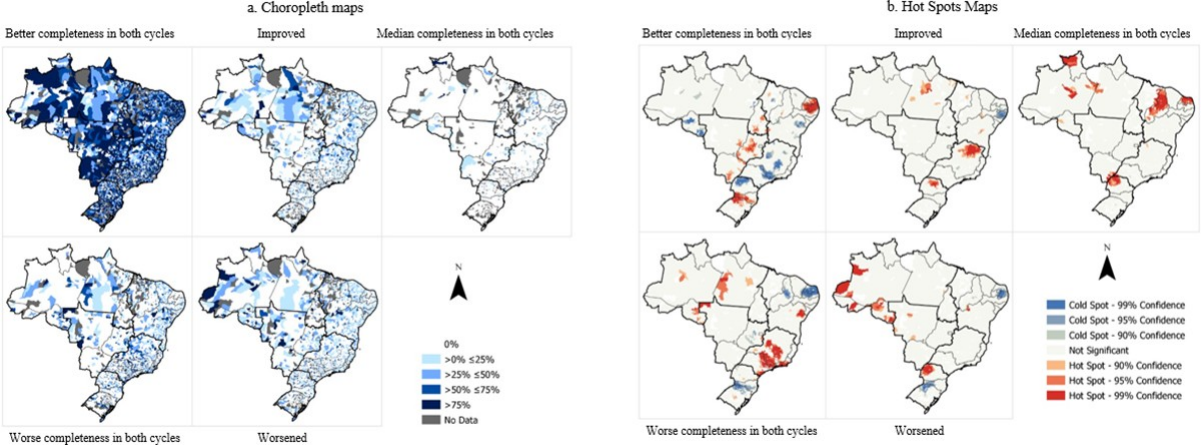
a. Choropleth maps of structure maps of PHC facilities, cycles 1 and 2 of PMAQ-AB, Brazil. b. Hot Spot maps of structure maps of PHC facilities, cycles 1 and 2 of PMAQ-AB, Brazil.

**Fig 2:**
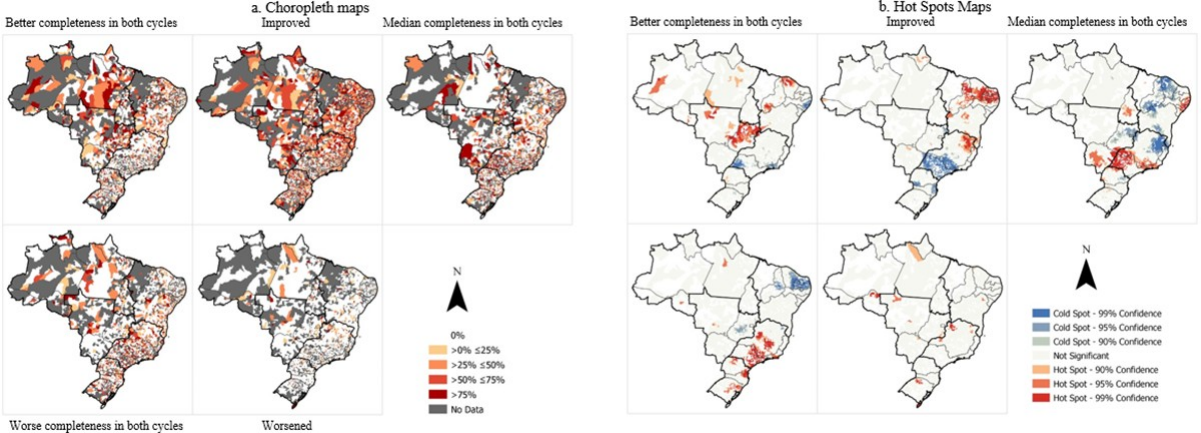
a. Choropleth maps of the work process of the PHC teams, cycles 1 and 2 of the PMAQ-AB, Brazil. b. Hot Spot maps of the work process of the PHC teams, cycles 1 and 2 of the PMAQ-AB, Brazil.

Spatial aggregation was conducted by municipalities, and they were classified on a 5-level scale: 0%, between 0 and 25%, above 25 and below 50%, above 50 and below 75%, and above 75% in each latent class (Figs 1 and 2). The hot spot analysis was also performed for each of the latent classes of the structure and the work processes to identify nearby areas with concentration of statistically significant “hot” (event increase) and “cold” spots (event decrease).

### Ethical considerations

The project was approved by the Ethics Committee of the University of Pelotas (UFPel), under the number of Letter 38/12 on May 10, 2012, in compliance with Resolution 196/96 of the National Health Council, in force at the time. All interviewed individuals signed the informed consent form.

## Results

Among the PHC facilities that participated in both Cycles I and II of the *PMAQ-AB*, the proportion of units with at least one dentists increased by 3.5%; the proportion of units that had one or more dentists, dental office, and operated for at least two shifts/day and five days/week increased by 2.4%. The work processes also underwent changes between the two cycles. The number of PHC teams that scheduled appointments every day of the week increased by 8.7%, and those who made home visits increased by 18.8%. However, there was a decrease in the number of teams that reported ensuring appointments for accommodating spontaneous demand (decrease of 4.2%), and in the continuity of treatment (decrease of 3.6%) (Table 1).

**Table 1:** Descriptive statistics of the variables used in latent transition analysis.

Indicators of latent status	Label	Time 1 frequency (%)	Time 2 frequency (%)	P-value*
**Oral health** **structure** **of the PHC facilities** (N = 23,021)				
Dentist	No	5659 (24.71)	5087 (22.10)	**<0.001**
	Yes	**17243 (75.29)**	**17935 (77.90)**	
Dentist + Dental office	No	6662 (29.14)	6910 (30.02)	0.040
	Yes	16199 (70.86)	16112 (69.98)	
Dentist + Dental office + office hours	No	7794 (34.44)	7568 (32.87)	**<0.001**
	Yes	**14838 (65.56)**	**15454 (67.13)**	
**Work process related to oral health in PHC teams (N = 15,699)**				
Scheduling the dental office every day of the week	No	9849 (62.74)	9337 (59.48)	**<0.001**
	Yes	**5850 (37.26)**	**6362 (40.52)**	
Performing basic procedures^1^	No	7506 (47.81)	7652 (48.74)	0.099
	Yes	8193 (52.19)	8047 (51.26)	
Performing home visit	No	8712 (55.49)	7397 (47.12)	**<0.001**
	Yes	6987 (**44.51)**	8302 (**52.88)**	
Organize the agenda to guarantee the attention to spontaneous demand	No	5843 (37.22)	6263 (39.89)	**<0.001**
	Yes	9856 (**62.41)**	9436 (**60.11)**	
Organize the agenda to ensure continuity of treatment	No	5744 (36.59)	6106 (38.89)	**<0.001**
	Yes	9955 (**63.41)**	9593 (**61.11)**	

* Chi-Square Test (α = 1%)

^1^Typical application of fluoride, amalgam restoration, composite resin restoration, tooth extraction, delay dressing/access to pulp or pulpotomy, dentoalveolar abscess drainage, scraping, straightening and supragingival polishing.

PHC: Primary health care.

Table 2 presents the model fit information used in the selection of final models in the present study. For both analyses, we selected three statuses models due to better fit properties and conceptual interpretability. The entropy of both models—PHC facilities structure (Entropy = 0.951) and the work processes of the teams (Entropy = 0.773)—was considered good.

**Table 2:** Model fit information used in selecting the LTA model.

	Number of Latent Classes	Likelihood Ratio Chi-Square	Degrees of Freedom	P-value	AIC	BIC	Entropy
Oral health **structure** of the PHC Facilities	2	409.16^2^	84	<0.001	77204.67	77277.07	
	**3**	**42.03**^1^	**44**	**0.557**	**74756.28**	**74893.03**	**0.951**
	4	409.16^2^	84	<0.001	75109.08	75326.27	
**Work process** related to oral health in PHC teams	2	6920.88	1010	<0.001	143123.85	143223.45	
	**3**	**4135.16**	**1000**	**0.001**	**74756.28**	**74893.03**	**0.773**
	4	3231.68	988	<0.001	139478.65	139746.80	

*Note*: Bold entries reflect the selected model.

^1^Chi-Square Test of Model Fit for the Binary and Ordered Categorical (Ordinal) Outcomes.

^2^Chi-Square Test for MCAR under the Unrestricted Latent Class Indicator Model.

PHC: Primary health care.

AIC: Akaike information criterion.

BIC: Bank identifier code.

The complete set of estimates of the parameters of the three statuses model of PHC completeness for the structure and work processes are presented in Tables 3 and 4, respectively. The top pane of the tables exhibits the probabilities of response to the item, depending on the latent status association. Notably, they equal during both the times (Cycles I and II), so these probabilities are identical for Time 1 and Time 2. The combination of probabilities of response to the item provides a notion of what characterizes the three different classes of completeness for the structure of the PHC facilities and for the work processes of the PHC teams.

**Table 3:** Three-status model of completeness and transition probabilities for the oral health structure of the PHC facilities in Brazilian PHC.

	Latent Status		
	Class 1	Class 2	Class 3
	Better completeness (Adequate oral health structure)	Median completeness (Dentist presence only)	Worse completeness (Lack of oral health structure)
**Probability of item response (class/status latent)**^**1**^:			
Have a dentist			
No	0.000	0.329	1.000
Yes	1.000	0.671	0.000
Have a dentist and dental office			
No	0.000	1.000	1.000
Yes	1.000	0.000	0.000
Have dentist, dental office and works at least at minimum time^2^			
No	0.056	1.000	0.999
Yes	0.944	0.000	0.001
**Prevalence of Latent Status:**			
Time 1 (Cycle 1 of PMAQ-AB, 2012)	0.710	0.044	0.246
Time 2 (Cycle 2 of PMAQ-AB, 2014)	0.670	0.134	0.166
**Transition probabilities** (Rows for Time 1, Columns for Time 2):			
Better completeness	**0.859**	0.108	0.033
Median completeness	0.537	**0.463**	0.000
Worse completeness	0.243	0.048	**0.708**

*Note*. Transition probabilities in bold font correspond to membership in the same latent status at both times.

^1^Item-response probabilities constrained to be equal at time 1 and time 2. These probabilities help us to understand the characteristics of the PHC facilitie that belong to the different latent classes/status.

^2^The PHC facilitie operated for at least two shifts/day (morning and afternoon) and five days/week (Monday to Friday).

^3^Based on their most likely latent class pattern.

PMAQ-AB: Program for Improvement of Access and Quality of Primary Care.

PHC: Primary health care.

**Table 4:** Three-status model of completeness and transition probabilities for the work process related to oral health in Brazilian PHC teams.

	Latent Status		
Probability of item response (class/status latent)^1^:	Class 1	Class 2	Class 3
	Better completeness (Adequate oral health process)	Median completeness (Biomedical model)^1^	Worse completeness (No oral health care offered)
Scheduling the dental office every day of the week			
No	0.371	0.500	0.994
Yes	0.629	0.500	0.006
Performing home visit			
No	0.044	0.589	0.955
Yes	0.956	0.411	0.045
Performing basic procedures^2^			
No	0.146	0.347	0.999
Yes	0.854	0.653	0.001
Attention to spontaneous demand			
No	0.052	0.155	0.998
Yes	0.948	0.845	0.002
Continuity of treatment			
No	0.030	0.152	1.000
Yes	0.970	0.848	0.000
**Prevalence of Latent Status:**^**3**^			
Time 1 (Cycle 1 of PMAQ-AB, 2012)	0.266	0.445	0.289
Time 2 (Cycle 2 of PMAQ-AB, 2014)	0.503	0.150	0.347
**Transition probabilities** (Rows for Time 1, Columns for Time 2):			
Better completeness	**0.903**	0.000	0.097
Median completeness	0.457	**0.361**	0.182
Worse completeness	0.105	0.070	**0.826**

*Note*. Transition probabilities in bold font correspond to membership in the same latent status at both times.

^1^Item-response probabilities constrained to be equal at time 1 and time 2.

^2^Typical application of fluoride, amalgam restoration, composite resin restoration, tooth extraction, delay dressing/access to pulp or pulpotomy, dentoalveolar abscess drainage, scraping, straightening and supragingival polishing.

^3^Based on their most likely latent class pattern.

PMAQ-AB: Program for Improvement of Access and Quality of Primary Care.

PHC: Primary health care.

The first latent status was called “better completeness” because all health facilities included in Class 1 were those with better structure—had *dentist* (100%), dental office (100%), and operated at least during the minimum office hours (94.4%). The second latent status, called “median completeness,” was characterized by health facilities that comprised only the professional (human resource component of the structure) but did not possess the equipment of the dental office. The third latent status was called “worse completeness.” In this class, the facilities did not have an adequate structure to perform dental care for the population (Table 3). Table 3 also provides the prevalence of each completeness profile in Time 1 and Time 2. The modal completeness profile was “better completeness” during both the time frames for the structure of health facilities; this profile comprised 71.0% and 67.0% of the sample in Time 1 and Time 2, respectively. The prevalence rates of the other two completeness profiles were not stable over time. The proportion of health facilities with median performance increased by 204% (from 4.4% in Time 1 to 13.4% in Time 2), while those with poor performance decreased by 32.5% (from 24.6% in Time 1 to 16.6% in Time 2). Diagonal elements for transition probabilities reflect the proportion of health facilities with the same profile of completeness during both the time frames. For example, in Time 1 “better completeness” of the structure had a probability of 0.859 of still being classified as “better completeness” in Time 2 (Table 3).

The PHC teams in Class 1 presented a better performance in the work processes (“better completeness”), i.e., they scheduled appointments for the dental office every day of the week (62.9%), conducted home visits (95.6%), performed all basic procedures (85.4%), guaranteed attention to spontaneous demand (94.8%), and guaranteed continuity of treatment (97%). “Median completeness” was characterized by the organization of the work processes according to a hegemonic biomedical model, since the percentages of the variables in this completeness indicate the practice of assistance by free demand in the office, limited scheduling offer for the dentist and predominantly curative. In the “worse completeness,” the work processes were not suitable for performing dental care for the given population, once the teams inserted in it, do not perform the evaluated procedures (Table 4).

The proportion of teams with “better completeness” increased by 89.1%, those with “worse completeness” increased by 20%, and those with “median completeness” decreased by 66.3%. The diagonal elements of the transition probabilities revealed that in Time 1, the “better completeness” status for the work processes had a probability of 0.903 of still being classified as “better completeness” in Time 2; the probability was 0.361 for “median completeness,” and 0.826 for “worse completeness” (Table 4).

The Choropleth maps describe the spatial distribution of latent transition patterns considered to evaluate the structure of health facilities and the work processes of the PHC team by FU (Figs 1A and 2A). The results indicate that the structure of the PHC facilities remained mostly with the status “best completeness” in both cycles in all states of the federation. North region comprised states with the highest percentage of improvement from Cycle I to Cycle II; however, in this same region, there are areas with highest percentage of health facilities that remained worse or worsened from Cycle I to II.

In the hot spot (Fig 1B), there is an accumulation of “hots” points indicating the presence of establishments with better completeness for the Midwest region of the country, the northeast of the Northeast and the extreme south of the country, and which improved the structure in Cycle II in the north of the North regions, Southeast and South. The North and Southeast regions also stand out for the presence of hotspots for establishments with worse completeness or that worsened in Cycle II.

The “better completeness” or “improved the process work” statuses were those with the highest percentage of teams in Brazilian municipalities. The North, Center-West and Northeast regions stand out for the presence of areas with no participating teams in both PMAQ-AB cycles, which resulted in the gray areas on map 2a. In the hotspot of the work process (Fig 2B), the Midwest, North and Northeast regions stand out with the status “best completeness” in both cycles. The Northeast of Brazil and the North of the Southeast region presented remarkable hotspots in improving the work process from Cycle I to Cycle II. In contrast, part of the Southeast region was covered with a coldspot, indicating a low number of teams with improvement in the work process, as can also be seen in areas of the South region. As for the “worst completeness” status, part of the Northeast region presented coldspot. On the other hand, hotspot areas appear in almost the entire Southeast and South regions of the country.

## Discussion

This is the first study that used latent class transition analysis to evaluate possible changes in potential access to oral health services, using data from two cycles of the *PMAQ-AB* related to the structure and work processed. The study’s hypothesis that the indicators of potential access in oral health improved between the first and second cycles of the program was corroborated by our results.

Regarding the structure, there was no increase in the proportion of units with a dentist, dental office, and operation during minimum office hours; it was considered that for those with better structure, the proportion of health facilities with median performance increased significantly (increase of approximately 204%), and for those with poor performance (did not have dentists and dental offices and did not operate during minimum office hours) the proportion of health facilities decreased by 32.5% in Cycle II.

For dentistry, the ability to provide good quality care depends on the availability of services in a timely manner [48], which invariably relies on the availability of human resources [49], and a structure for performing clinical procedures. Therefore, in this study, the evaluation of the structure of oral health services focused on verifying these attributes that were considered to be essential.

The inclusion of oral health teams in the *FHS* leads to an effective dental practice [48], which presupposes the presence of oral health professionals close to people to ensure the establishment of bond and accountability over a given territory and population. Thus, precarious conditions and inadequacies related to the structure of oral health facilities may cause limitations and difficulties in the use of these services by the community [5, 50]. With these limitations, human resources management is considered an essential factor in the provision of quality health services [49, 51].

In addition, studies have revealed that the lack of oral health professionals compromises the work performed in the primary care and limits the access of users [52, 53], increasing the demand in hospitals, specialized services, and in urgency and emergency networks due to the low resolvability of this level of care, which does not satisfy the PHC principle of functioning as a gateway to the health system and being an organizer of health care networks [51].

For the team’s work processes, only two indicators (“organize the agenda to ensure a response to spontaneous demand” and “continuity of care”) exhibited a decrease in Cycle II. Still, the work processes had a satisfactory performance, with an increase in the proportion of teams belonging to the status “best completeness” in Cycle II.

How to schedule appointments can be considered a factor that enables the individual to use health services directly related to potential access, thus acting as a factor that limits or expands the individual’s ability to use these services [26, 36]. In this study, there is an increase in the number of PHC teams that perform full-time scheduling in both shifts and five days a week; this aspect shows a possible expansion of access to oral health services, and it agrees with other studies that indicate that improvements in the scheduling model may increase access to PHC [5, 37, 54].

The percentage of PHC teams performing all basic procedures, which evaluates the performance of curative and preventive actions aimed at preventing and/or repairing the main diseases in oral health and promoting health, was the only indicator related to the work processes that did not present a statistically significant difference when we compare the two cycles of the *PMAQ-AB*.

In a study that assessed the association between sociodemographic aspects, indicators of the health system in Brazilian municipalities, and characteristics of the work process and the performance of a list of curative dental procedures by the oral health teams evaluated during the first cycle of the PMAQ-AB, the authors identified that the prevalence of performing curative dental procedures (the same analyzed in our study, except for topical fluoride application) was 69.51% and that 30.49% of oral health teams do not perform one or more of the dental procedures, indicating the need for changes in the oral health care model [55].

Baumgarten et al. (2018) identified that only 14.8% of the 18,114 OHT evaluated offered basic clinical procedures, considering the presence of a minimum set of dental equipment, instruments and supplies, emphasizing the need for improvements in the existing structure to ensure quality dental care in APS.

Although it is perceived that teams still operate according to traditional logic with a focus on therapeutic and curative actions [48, 55], there are advances, such as home visits, that are configured as a tool in the implementation of initiatives to change the work processes; this aspect indicates that this strategy is a determinant to strengthen the teams’ bond with the family, favoring a closer relationship between the population and health services [19]. In this study, a home visit was one of the actions that also showed strengthening with the implementation of *PMAQ-AB*, demonstrated by the percentage increase in the number of teams that rescued the practice of this action, strongly guided by the FHS.

Although the decrease in the number of teams that guarantee scheduling to continuity of treatment in Cycle II identified in our study shows the presence of failures in the work processes of PHC teams that compromise the supply of services and limit their utilization by users [35, 56], it is considered that this data indicate progress in the qualification of oral health services related to the longitudinal aspect of care; this is one of the premises of the FHS, as more than 60% of the teams are guaranteeing an agenda for continuity of care in both the cycles.

It is known that the development of actions that guarantee the accommodating of spontaneous demand and continuity of care fosters a bond and accountability between professionals and users and thus qualifies access to oral health care. However, the difficulty in coordinating these activities with those planned by the service may cause the service supply to be inadequate for the needs of the users, imposing limitations on the implementation of a joint planning that recognizes the complexity of the epidemiological framework and ensures the continuity and the longitudinal aspect of care [57].

Fagundes et al. (2018), in a study that described the work process of Brazilian oral health teams, based on the essential attributes of PHC, identified a low percentage of teams carrying out actions for the coordination of care, which include actions to guarantee the continuity of treatment.

Other studies have revealed lacunas in the work process of the teams [26, 35, 51, 58], but our research identifies alarming data, i.e., the increase in the prevalence of PHC teams with “worse completeness” in Cycle II, representing an increase in the proportion of teams that do not perform any of the work processes indicators surveyed, is considered a serious barrier to potential access to oral health services.

A possible explanation for this problem could be related to the performance evaluation model that was used by *PMAQ-AB*, which intended the transfer of financial incentives to teams with better performance, thereby not encouraging the change in behavior of teams considered to be “worse completeness.”

Although the number of PHC teams deployed in Brazil has increased between cycles, oral health coverage has changed little, which may contribute to the persistence of problems in accessing services, such as those perceived in this study, related to the lower possibility of immediate care or for continuity of treatment, and also a reduced offer of clinical procedures.

In the spatial analysis of the latent transition pattern, the structure of health facilities was predominantly presented with an adequate structure (“better completeness”) in all *FU*. The north region, however, has several characteristics, presenting the highest percentages of facilities with “better completeness,” while in the same region, there are *FU* that have the highest percentages of facilities with an inadequate structure for providing oral health services. However, still in this analysis, the north region presented the highest frequency of teams with “better completeness” for the work processes and, together with the northeast region, the highest frequency of teams that improved indicators in Cycle II, reflecting greater socioeconomic disparities and inequalities in the health care domain in Brazil [7, 26].

Still, in relation to the work process, the southeast and south regions revealed the predominant FU with the worst transition patterns (“worse completeness”/worse); this finding is in line with the results that have been found in other studies, which indicate that the regions with the best socioeconomic indicators would be those that provide a higher quality of services to the population [19, 35, 53].

The different patterns found between regions can be justified by the fact that improvements related to the structure of health facilities require financial contributions from municipal and state management, while adequacy of the team’s work processes involves actions more focused on aspects related to governance, organization, and planning that can be easily implemented.

As the FU that presented greater difficulty in improving their standards regarding the structure of oral health services are precisely those that are located in the region with the worst socioeconomic indicators in Brazil, it is understandable that these states encounter financial difficulties in implementing such improvements.

Besides, the conditions of the structure of services may have a direct impact on the work processes of the PHC team, but they do not guarantee an increase in the rate of use or an improvement in the quality of care provided [7, 8].

It is also necessary to consider some limitations of this study. The use of secondary data limits the study analysis to existing data. Also, some access variables could not be evaluated because they were not collected during the two cycles of the program, thereby making comparisons impossible.

Data referring to the work process module in both cycles of the *PMAQ-AB* were sampled by self-adherence. There are indications that the best-structured teams were those who joined the program [51], thus inflating the results. However, adherence during Cycle II increased significantly, including units with more considerable variability, and therefore better reflecting the local reality of the health facilities evaluated. Thus, the identification of improvement in indicators reflects, in fact, changes in the structure and work processes in the primary care domain.

The study is subject to measurement bias, as the information was collected through interviews. However, this bias was minimized as the collection took place on the spot, allowing the interviewer to check the items in the structure. In addition, some questions regarding the work process require documentary proof.

The results of this study may guide the implementation of measures aimed at expanding potential access, as well as improving the quality of oral health services offered in Brazil. The application of LTA is the unique aspect of our study, as it allowed us to evaluate the behavior of access indicators over time through two evaluations conducted by *PMAQ-AB*. However, it is recommended that further studies be carried out taking into account the PMAQ-B cycle III data, in order to identify a possible trend in the behavior of these indicators.

Therefore, the establishment of evaluation processes may be related to positive changes in indicators related to access to oral health services, increasing the capacity of users to potential access. However, some attributes of access remain unsatisfactory and are persisting organizational barriers, such as scheduling, ensuring continuity of care for users, and limited availability of curative and preventive procedures.
